# Complete genome sequence of a putative novel orthotospovirus species identified in *Capsicum* fruits from South Africa

**DOI:** 10.1128/mra.00561-25

**Published:** 2025-09-08

**Authors:** Marcel Westenberg, Christel de Krom, Carla Oplaat, Jerom van Gemert, Annelien Roenhorst, Ruben Schoen, Pier de Koning, Marleen Botermans

**Affiliations:** 1 Netherlands Institute for Vectors, Invasive plants and Plant health (NIVIP), National Plant Protection Organization (NPPO), Netherlands Food and Consumer Product Safety Authority (NVWA) 84859 , Wageningen, the Netherlands; Katholieke Universiteit Leuven, Leuven, Belgium

**Keywords:** *Capsicum* sp., orthotospovirus, South Africa, high-throughput sequencing, putative novel species

## Abstract

We report two complete genome sequences of a putative novel orthotospovirus species in pepper fruits (*Capsicum* sp.) from South Africa, provisionally named *Orthotospovirus capsici* (Capsicum orthotospovirus 1; CaV1). Its nucleocapsid protein shows less than 88% amino acid identity with other orthotospoviruses.

## ANNOUNCEMENT

Orthotospoviruses (genus *Orthotospovirus*, family *Tospoviridae*) are enveloped, quasi-spherical viruses with a tripartite negative and ambisense RNA genome, encoding five open reading frames (1). Many cause significant crop losses worldwide, affecting both yield and quality (2).

**Fig 1:**
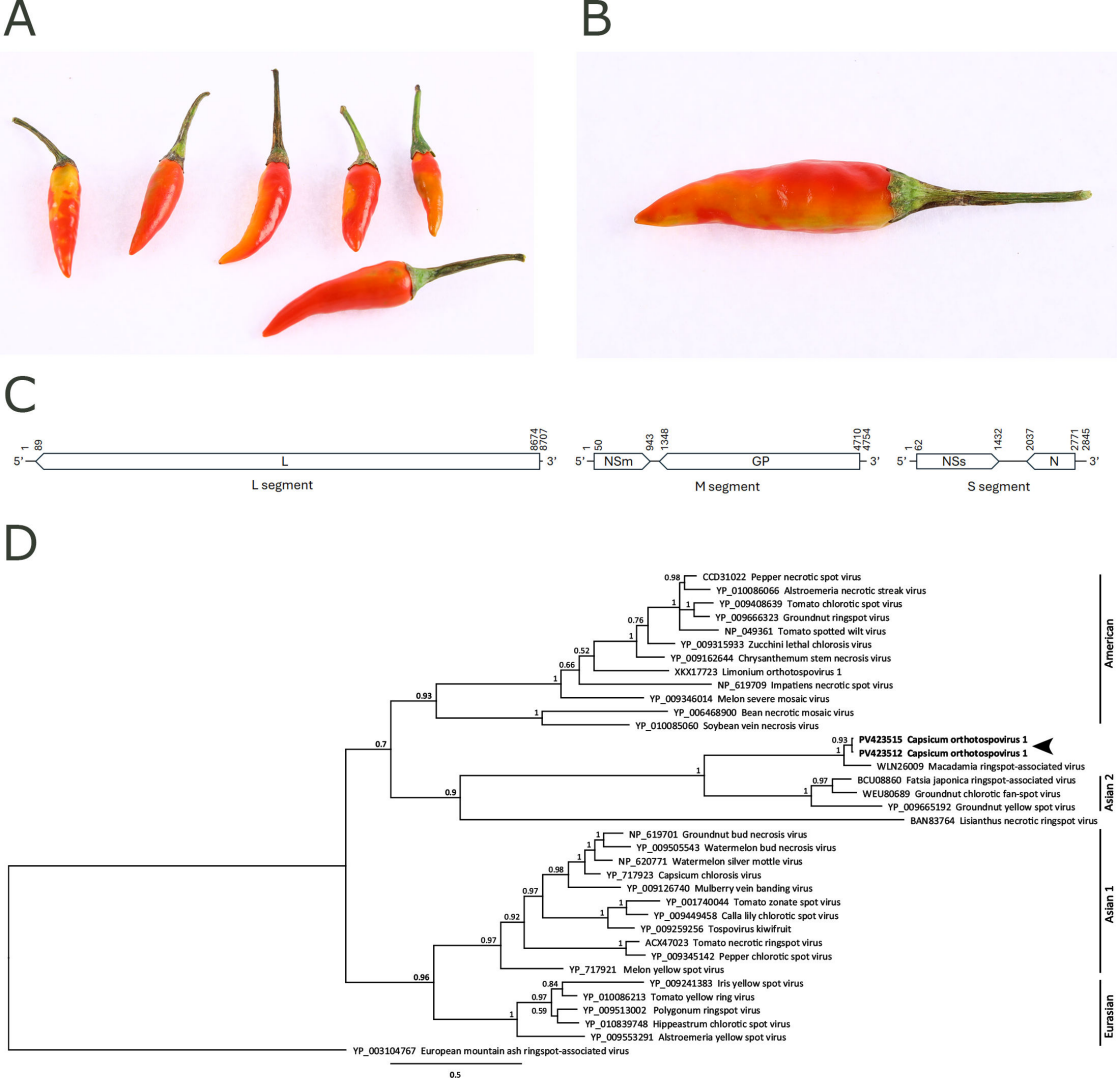
Symptoms on *Capsicum* sp. fruits infected with Capsicum orthotospovirus 1 (CaV1) and other viruses, its phylogenetic relationship with other orthotospoviruses, and its genome architecture. (**A, B**) Fruits with discolorations—irregular yellowish spots, sometimes with a dark margin—in samples NPPO-NL 36567563 (**A**) and NPPO-NL 40782061 (**B**). (**C**) Genome architecture of CaV1. Nucleotide numbers for isolate NPPO-NL 36567563 are indicated at the start and end of each genome segment and predicted open reading frames. L: RNA-dependent RNA polymerase; NSm: non-structural protein (cell-to-cell movement); GP: glycoprotein precursor; NSs: non-structural protein (silencing suppressor); N: nucleocapsid protein. (**D**) Bayesian phylogenetic tree of Orthotospovirus N protein amino acid sequences made in Geneious Prime 2025.0.3 (Biomatters, New Zealand). Sequences were aligned using MAFFT (4), and the tree was constructed with MrBayes V3.2.6 (5) applying the WAG model of amino acid substitution with a gamma distribution to account for rate variation. MrBayes was run with four chains for 1.1 million generations, sampling trees every 200 generations. The first 100,000 trees were discarded as burn-in. The tree is rooted with European mountain ash ringspot-associated virus as an outgroup. Posterior probabilities are indicated at the nodes, and the distance bar represents 0.5 amino acid substitutions per site. CaV1 is indicated with an arrowhead. Major clades are marked on the right.

In June and July 2021, *Capsicum* fruits from two South African consignments displaying virus-like symptoms (Fig. 1A and B) were submitted to the Netherlands Institute for Vectors, Invasive plants and Plant health by phytosanitary inspectors to investigate potential viral infections and assess phytosanitary risks. These samples, comprising six (NPPO-NL 36537563) and one fruit (NPPO-NL 40782061), were analyzed at different time points using high-throughput sequencing as previously described (3).

For both samples, symptomatic tissue was collected from each fruit up to 1 g for total RNA extraction using the RNeasy Plant Mini Kit (Qiagen, the Netherlands). Ribosomal RNA was depleted with the Ribo-Zero rRNA removal Kit (Illumina), followed by library preparation with the NEBNext Ultra II Directional RNA library Prep Kit (New England Biolabs, USA). The libraries were sequenced on an Illumina NovaSeq 6000 (150 nt paired-end reads).

The reads (24,741,042; 36,239,122) were quality scored (HQ30 >95%), trimmed (remaining reads: 24,740,450; 36,238,830), and *de novo* assembled using CLC Genomic Workbench (v21.0.4 [Qiagen]) standard tools and default settings. Consensus sequences (>100 nt; read depth >10) from the *de novo* assemblies were analyzed using MegaBLAST and DIAMOND with default settings (6) with a locally installed NCBI nr (/nt) database (download 05 July 2021). Visualizations of BLAST results were performed in Krona with a bitscore threshold of 25 (7). For each sample, various contigs showed significant similarity to viral genomes, including sequences from orthotospoviruses. These orthotospovirus contigs corresponded to the three genome segments L, M, and S, together encoding five open reading frames (Fig. 1C; Table 1). Rapid amplification of cDNA ends (RACE) was not performed, as the conserved inverted repeats in the terminal nine nucleotides indicated segment completeness (8). Segment length differences are due to indels in non-coding regions; nucleotide identity between genomes is 99.5%.

**TABLE 1:** Viral sequence information per sample

Sample	Segment	Length (nt)	GC content (%)	Mean read depth
NPPO-NL 36537563	L	8,707	34	7,804
	M	4,754	35	13,754
	S	2,845	35	33,817
NPPO-NL 40782061	L	8,706	34	34,836
	M	4,753	35	83,118
	S	2,842	38	106,142

The nucleocapsid (N) protein from the S segment shares 87.8% amino acid identity with that of macadamia ringspot-associated virus (MRSV), which is below the 90% species demarcation threshold (1), supporting the classification of this virus as a novel species, provisionally named *Orthotospovirus capsici* (Capsicum orthotospovirus 1; CaV1). A Bayesian tree (Fig. 1C) based on N protein sequences placed CaV1 as sister to MRSV from South African macadamia trees (9). Together, they form a sister group to the “Asian 2 clade”. Given the presumed shared geographic origin, future discoveries may support the recognition of a distinct clade with an African lineage.

Besides CaV1, potato virus Y and pepper cryptic virus 1 were detected in both samples. Sample NPPO-NL 36537563 also contained a polerovirus from the pepper vein yellows group, pepper vein yellows virus-associated RNA, pepper cryptic virus 2, and a tentative novel emaravirus. Although irregular spots on *Capsicum* fruits have been associated with orthotospoviruses (10), and both potato virus Y and poleroviruses are linked to discolorations (11, 12), the role of CaV1 in the observed symptoms remains unclear due to the mixed infections.

## Data Availability

The complete genome sequences has been deposited at GenBank under accession numbers: PV423510, PV423511 and PV423512 for isolate NPPO-NL 40782061, and PV423513, PV423514 and PV423515 for isolate NPPO-NL 36567563. The raw reads were deposited under SRA accession number SRX28180310 and SRX28180311 for isolate NPPO-NL 36567563 and NPPO-NL 40782061, respectively.
